# Primary Paraspinal Leiomyosarcoma in a 49-year-old Woman: Index Case from Pakistan

**DOI:** 10.12669/pjms.41.13(PINS-NNOS).13501

**Published:** 2025-12

**Authors:** Haseeb Mehmood Qadri, Shariqa Batool, Raahim Bashir, Shafqat Ali, Ahtesham Khizar

**Affiliations:** 1 Haseeb Mehmood Qadri, MBBS, Punjab Institute of Neurosciences, Lahore, Pakistan; 2 Shariqa Batool Medical Student, Aga Khan University Hospital, Karachi, Pakistan; 3 Raahim Bashir, MBBS, Punjab Institute of Neurosciences, Lahore, Pakistan; 4 Shafqat Ali, MBBS, Punjab Institute of Neurosciences, Lahore, Pakistan; 5 Ahtesham Khizar, MBBS, FCPS Consultant Neurosurgeon & Senior Registrar, Punjab Institute of Neurosciences, Lahore, Pakistan

**Keywords:** Leiomyosarcoma, Primary, Thoracic vertebrae, Pakistan

## Abstract

Leiomyosarcoma is a rare and aggressive malignant neoplasm arising from smooth muscle cells, commonly affecting the uterus, gastrointestinal tract, or retroperitoneum, with paraspinal involvement being extremely uncommon. Clinical symptoms include back pain, weakness, and sensory deficits due to spinal cord compression.

We present a 49-year-old woman with bilateral lower extremity weakness for 15 days, lower back pain, and a left paraspinal swelling for two months. Examination showed a tender lumbar paraspinal mass, paralysis below T10, and saddle anesthesia. MRI with contrast showed large left paraspinal soft tissue mass, confirmed as grade 2 leiomyosarcoma on biopsy. Complete tumor excision was done, and postoperative recovery was uneventful.

This report highlights an unusual presentation of primary lumbar paraspinal leiomyosarcoma without regional or distant metastasis in a middle-aged woman, emphasizing the importance of thorough evaluation to exclude potential primary sites. Diagnosis relies on biopsy with immunohistochemistry, while surgical excision remains the mainstay of treatment.

## INTRODUCTION

Leiomyosarcoma is a rare malignant neoplasm of smooth muscle origin and is most commonly encountered in the uterus, gastrointestinal tract, and retroperitoneum.^1^ About 7% of all soft tissue sarcomas are diagnosed as leiomyosarcoma and are usually secondary to spinal metastasis in the setting of a primary source.^2^ Primary paraspinal leiomyosarcoma is an exceedingly rare entity with nonspecific radiological and clinical features that are often indistinguishable from far more prevalent pathologies such as spinal metastases.^3^ This necessitates histopathological confirmation acquired by biopsy. Immunohistochemical staining for smooth muscle actin and desmin is reliably specific for diagnosis.^2^

Although previous reports of paraspinal leiomyosarcomas have been documented, these cases have usually been in the cervical spine with significant vertebral involvement.^4^ Here we present a rare presentation of a lumbar primary paraspinal leiomyosarcoma in a middle-aged woman. Accurate diagnosis includes exclusion of primary sources and histopathological confirmation obtained through biopsy, with the goal of treatment being complete resection. Clinicians should be cognizant of primary leiomyosarcoma when encountering an isolated paraspinal mass.

## CASE PRESENTATION

**Fig.1:**
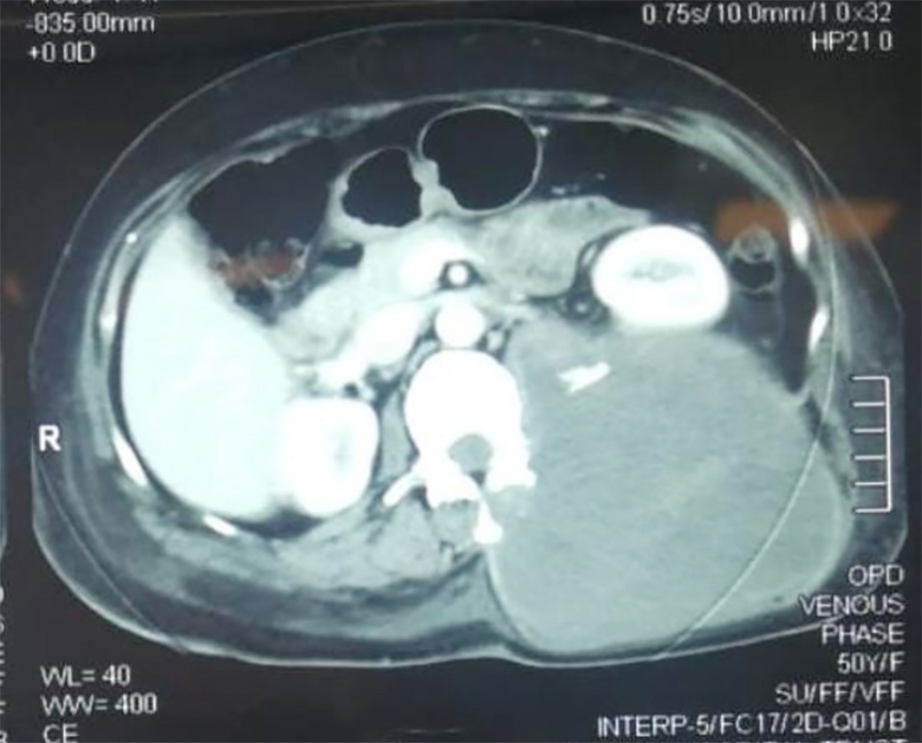
MRI with contrast showing large soft tissue mass measuring 99 × 96 mm in the left posterior paravertebral compartments at the lower lumbar region (yellow arrow).

A 49-year-old woman presented to the outpatient department (OPD) at the Punjab Institute of Neurosciences in June 2024 with complaints of bilateral lower limb weakness for the past 15 days, lower back pain for the past two months, and swelling in the left paraspinal region for the past two months. The onset of symptoms was slow and progressive in nature, associated with nausea and weight loss. On clinical examination, the patient had a swelling in the left lumbar paraspinal region, measuring approximately 10 × 10 cm. The swelling was tense and tender. Motor power in the lower limbs was 0/5, with a sensory level at the T10 vertebra, and saddle anesthesia was also present. Examination of the upper limbs was unremarkable. Based on the history and clinical examination, the differential diagnoses included metastatic disease, sarcoma, and abscess. Magnetic resonance imaging (MRI) with contrast revealed a large soft tissue mass measuring 99 × 96 mm in the left posterior paravertebral compartments at the lower lumbar region, suggestive of a neurogenic tumor (Fig.1). Further metastatic workup included computed tomography (CT) of the neck, chest, abdomen, and pelvis, which showed no metastatic lesions except for a large neoplastic mass in the left paraspinal region (12.4 × 12.2 × 19.2 cm). After investigations, the differentials included malignant nerve sheath tumor and soft tissue sarcoma.

**Fig.2:**
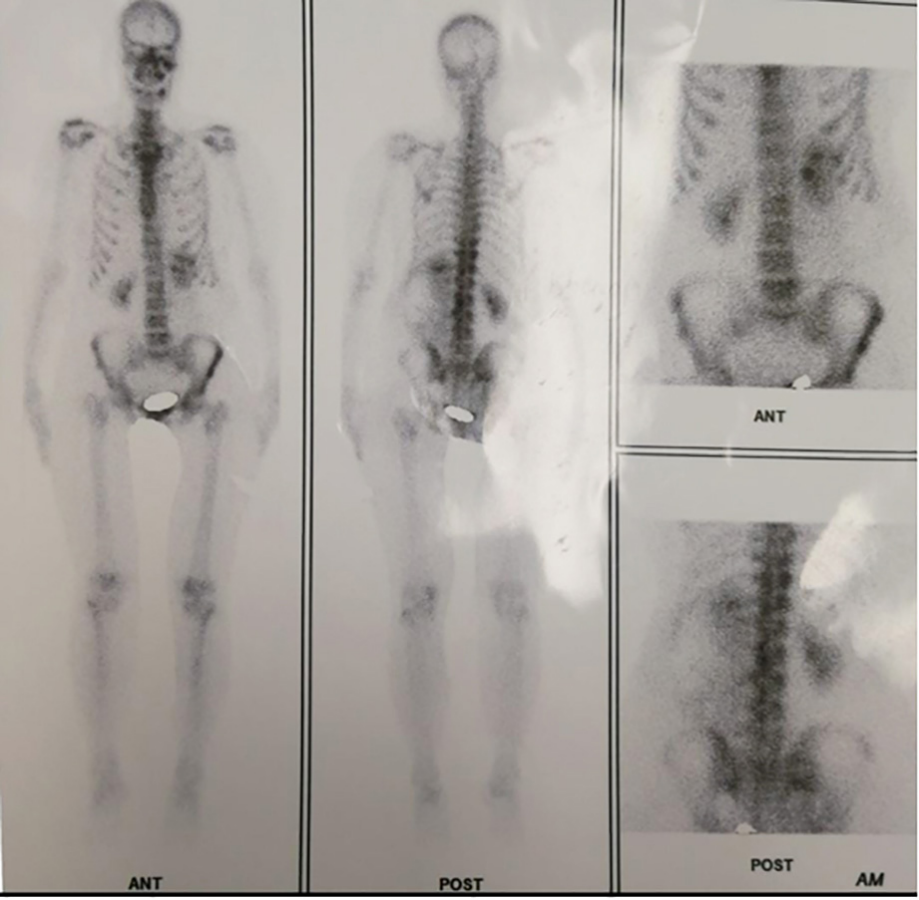
PET CT scan findings favouring soft tissue mass in left paraspinal region causing erosion of the 12th rib and adjoining transverse process of L1-2 vertebra.

**Fig.3:**
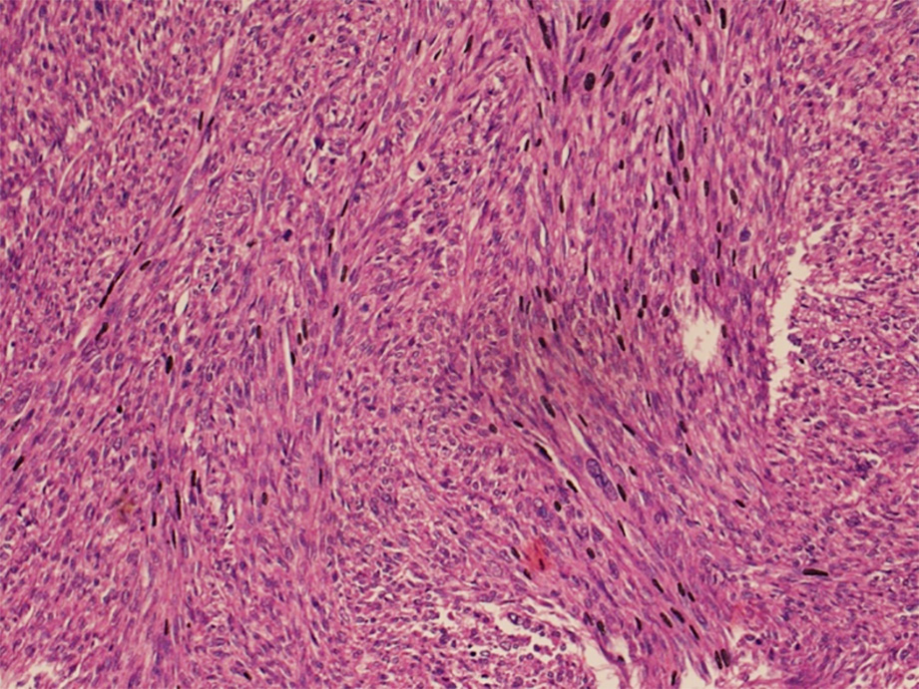
Histopathology under light microscope with hematoxylin and eosin (H & E) stains. Section reveals smooth muscle cells arranged in vague whorling pattern. The nuclei show marked pleomorphism and the section reveals high mitotic activity.

Subsequent workup included Positron Emission Tomography (PET) scan, which demonstrated a soft tissue mass in the left paraspinal region with erosion of the 12th rib and adjoining L1-2 vertebrae no primary source was noticeable from the usual sites of occurrence (Fig.2). Ultrasound (USG)-guided biopsy confirmed the diagnosis of Leiomyosarcoma, French Fédération Nationale des Centres de Lutte Contre le Cancer (FNCLCC) grade 2.^5^ (Fig.3).

Patient was scheduled for surgery in elective operation theatre. Under general anesthesia, intraoperative findings revealed a greyish, highly vascular tumor attached to adjacent structures. All tumor tissue was successfully excised, and hemostasis was secured. A closed-drain system was placed in the cavity. There were no intraoperative complications, and recovery from general anesthesia was smooth. The patient was transferred to the intensive care unit (ICU) for monitoring and later to the female ward. She had an in-hospital stay of two days. The drain was removed, and the patient was discharged with follow-up guidelines.

Histopathological evaluation of the resected trucut biopsy confirmed Leiomyosarcoma, FNCLCC Grade 2. Immunohistochemistry showed positivity for SMA, while staining for desmin, S100, and CD31 was negative. Upon follow-up, the patient returned to the OPD with complaints of pus discharge from the wound. She was admitted, and the wound was explored, washed, and pus was drained in the emergency operation theatre. Recovery from anesthesia was smooth, and the patient was shifted to the ward. Antibiotics were started, and the pus was sent for culture and sensitivity testing.

The patient was referred for adjuvant therapy to another hospital and was lost to follow up for the second clinic appointment afterwards.

## DISCUSSION

Leiomyosarcomas are considered rare malignant neoplasms accounting for about seven percent of the soft tissue sarcomas and less than 0.7% of primary malignant neoplasms which are aggressive in nature and associated with poor prognosis.^1^ Although leiomyosarcoma can arise from any site involving smooth muscle cells such as uterus, gastrointestinal tract or retroperitoneum, leiomyosarcoma of the spine and paraspinal region is extremely rare and most common site of involvement is the thoracic vertebrae and nearby region.^6^ Moreover, osseous metastatic lesions are rare with spine being the common location resulting in spinal cord compression with associated symptoms.^6^ Leiomyosarcomas in general are large volume tumors with irregular boundaries, moderate to severe vascularity and significant mass effect or adhesion to nearby structures.^4^

Leiomyosarcomas have a female preponderance (69.2%) and average age of presentation is around fifth to sixth decades of life which is almost in concordance with our case of 49 years old female.^2^ The majority cases in females may be attributed to the fact that some metastatic spinal leiomyosarcomas have primary uterine cancer origin or the high rate of smooth muscle proliferation due to estrogen effect but our case was unique with paraspinal region being the primary location and no signs of distant metastasis as confirmed by PET CT.^3^ A review of the literature is shown in Table-I below.

**Table-I:** Overview from 1992-2016 of the previously reported cases on primary paraspinal leiomyosarcoma.

Study by	Age	Gender	Symptoms at presentation	Signs at presentation	Site of lesion	Vertebral level of lesion	Opted management	Outcome
Gupta et al. 2016^3^	34	female	Low backache	radicular pain in the right lower limb	right paraspinal mass	L4-L5	Surgery + external beam radiation therapy + chemotherapy	Metastatic disease at 6 months
Sengupta and Nag 1992 7	43	male	Neck pain	no deficits	Right lateral musculature	C7-T1	Surgery + radiotherapy	4 years follow-up, no recurrence
Lehman et al. 2007 8	45	male	Painful enlarging mass on right side of neck	no deficits	right posterolateral musculature	C1-C2	Surgery + chemotherapy + radiotherapy	6 years-no primary site recurrence. Metastases present
Marshman et al. 2005 9	61	female	Cervical myelopathy	no deficits	posterior paraspinal musculature and ligamentum flavum	C3-C5	Surgery	Lost to follow-up
Aksoy et al. 2002 10	71	female	Pain in the neck and right leg	sensory loss on the right side of body	right paravertebral mass	T1-T3	Surgery + chemotherapy	Not known

Clinical manifestations and severity of these lesions depends on the location & grade of tumor and size & depth of the lesion.^3^ The frequent clinical presentations include focal neurological pain with associated weakness, numbness, restricted movements, and paraesthesia of the extremities. The onset of symptoms is usually slow and progressively worsening in nature over the course of months and our patient concurrently had worsening symptoms over period of four months resulting in saddle anesthesia.^1^ A case report by Aksoy et al^10^. revealed that back pain proved to be the most common presenting symptom (60.9%) followed by leg weakness (17.4%), numbness (13.0%), gait disturbance (8.7%) and paraesthesia (4.3%) all of which were consistent with findings in our patient.^2^ Amongst spinal level involvement, thoracic vertebrae have higher incidence of 46.9% followed by cervical (21.9%) and thoracolumbar (18.8%) while our patient had soft tissue mass eroding the twelfth rib and L1-L2 vertebrae as revealed by PET scan.

Both CT and MRI are unable to differentiate between neurogenic tumors and leiomyosarcomas which can be challenging in terms of diagnosis and treatment strategies to be employed.^4^ However, the tumor shape, boundaries, density, and location can be detected via these imaging modalities and our case also revealed a soft tissue mass measuring 99 × 96 mm in the left posterior paravertebral compartments at the lower lumbar region. As our initial differential included a nerve sheath tumor, a leiomyosarcoma presents on MRI as isointense on T1-weighted images and hyperintense on T2-weighted images with homogenous enhancement on contrast.^3^ Thus, the imaging manifestations showed some specificity according to the typical presentation of leiomyosarcomas in other common tumour sites.

Tissue biopsy is regarded as gold standard for detecting leiomyosarcoma and an ultrasound guided biopsy of the lesion was done for our patient confirming FNCLCC grade 2 Leiomyosarcoma after all distant metastases were ruled out on CT neck, chest, abdomen and pelvis.^4^ The disease prognosis is greatly determined by the size and histological tumor grade.^4^ Surgical resection of the tumor is the mainstay of treatment to provide symptomatic relief to patients but may fail to provide complete cure as leiomyosarcomas are particularly resistant to any post-operative chemotherapy and radiotherapy, while also having a greater rate of short-term recurrence and subsequent death.^2^,^4^

No literature consensus exists on treatment protocols and no randomized trials on leiomyosarcoma have been conducted so far.^3^ An approach for aggressive excisional surgery is required as it contributes to a much favourable prognosis with an overall one-year and five-year survival of 64% and 21% respectively.^6^ Our patient also had a successful excision of all tumor tissue. The use of total en bloc spondylectomy has also been considered which not only improves performance scale but also prolongs patient survival. 6 The use of adjuvant chemotherapy or post-operative radiotherapy remains controversial, and no such considerations were done for our case.^1^,^4^

A letter by Gupta et al. has shown that a local disease control may be achieved by postoperative external beam radiotherapy or low dose brachytherapy, but these tumors still have a median progression free survival of 1.5 years and overall survival of 7.1 years despite extensive measures.^3^ Patients generally have minimal post operative complications with improvements in symptoms such as back pain owing to the elimination of mass effect of the tumor. Our patient has no major complications with only two days hospital stay post procedure and was only managed for pus discharge from the wound. Since the study is on a rare disease, this vastly limited the number of cases we could analyse for comparison and apply to our case with a different demographic setting but the imaging findings aligned with typical leiomyosarcoma characteristics.

## CONCLUSION

The study presents an extremely rare presentation of leiomyosarcoma of the paraspinal region of primary origin without any regional or distant metastasis. Thorough investigation of potential primary sites needs to be done for adequate identification. This case shows both typical and atypical features of paraspinal leiomyosarcoma, but some findings have no plausible theory in existing literature. This case further reiterates that tissue biopsy and immunohistochemical staining are essential for the confirmed diagnosis of paraspinal leiomyosarcoma. It is also evident that a gross total resection confers improved long-term survival with significantly reduced recurrence and relief of symptoms secondary to compression.

### Authors’ Contribution:

**HMQ:** Supervision of the project, concept of work and critical review of the manuscript.

**SB, RB & SA:** Analysis and interpretation of data, drafting of the manuscript.

**AK:** Acquisition of data, critical review of the manuscript.

All the authors agree to final approval of version to be published and to be accountable for all the aspects of the work.
